# Comparison of Fetomaternal Outcomes in Patients With Gestational Diabetes Mellitus Treated With Insulin Versus Acarbose: Results of a Prospective, Open Label, Controlled Study

**DOI:** 10.7759/cureus.12283

**Published:** 2020-12-25

**Authors:** Suryakanta Jayasingh, Saumya Nanda, Sujata Misra, A Baliarsinha, Sidhartha Das, Anant Patil

**Affiliations:** 1 Obstetrics and Gynecology, Institute of Medical Sciences and SUM Hospital, Bhubaneswar, IND; 2 Obstetrics and Gynecology, Srirama Chandra Bhanja Medical College, Cuttack, IND; 3 Obstetrics and Gynecology, Fakir Mohan Medical College, Balasore, IND; 4 Endocrinology, Srirama Chandra Bhanja Medical College, Cuttack, IND; 5 Internal Medicine, Srirama Chandra Bhanja Medical College, Cuttack, IND; 6 Pharmacology, Dnyandeo Yashwantrao Patil University-School of Medicine, Navi Mumbai, IND

**Keywords:** acarbose, fetal outcomes, gestational diabetes, insulin

## Abstract

Objective

To evaluate fetomaternal outcomes in patients with gestational diabetes mellitus (GDM) treated with insulin versus acarbose.

Material and methods

In this prospective, open label, controlled study, GDM patients treated with insulin or acarbose were observed till six weeks after delivery. Maternal outcomes, fetal outcomes and glycemic control were compared between two groups.

Results

Fifty patients in each group (insulin group-mean age 28.52 years; acarbose group-mean age 26.26 years; p=0.020) were included. There was no difference in body mass index (p=0.157), family history of diabetes (p=0.648), history of GDM (p=0.50) or mean gestational age at diagnosis (p=0.245) between the two groups. There was no significant difference in the incidence of recurrent infections (p=0.64), pre-eclampsia (p=0.64) or premature rupture of membranes (p=0.40) between the two groups. Mean duration of gestational weeks at the time of delivery in the insulin and acarbose group was 36.93 and 38.36 weeks respectively (p=0.002). There was no difference in the modes of delivery, mean post-operative random blood glucose (p=0.96), fasting blood glucose level at day seven (p=0.15) and after six weeks (p=0.83) between the insulin and acarbose groups. There was no difference in reduction in the postprandial blood glucose level at day seven (p=0.48) and after six weeks (p=0.23). There was no significant difference in the mean birth weight of babies born to mothers treated with the two drugs (p=0.21). There was no difference in the incidence of neonatal complications between the two groups.

Conclusion

Acarbose can be an effective and well tolerated option for treatment of gestational diabetes mellitus.

## Introduction

Gestational diabetes mellitus (GDM) is a condition of glucose intolerance observed during pregnancy. It occurs due to defects in the response by the beta cells of pancreas to insulin during pregnancy and is one of the known common complications during pregnancy [1-4]. According to the results of a meta-analysis, overall prevalence of GDM in Europe is 5.4% [5]. Overall prevalence of GDM in different countries ranges from less than 2% to 17%. The rates differ between geographical areas and the type of population being studied. The literature also suggests rising rates of GDM [2]. Incidence of GDM is increasing worldwide [6].

Women with a history of GDM have a significantly higher risk of developing type 2 diabetes mellitus as compared to those without GDM [1,7]. Similarly, risk of metabolic syndrome is higher in patients with GDM than those without it [7]. Diagnosis and treatment of GDM is important to avoid maternal and fetal complications [8,9].

Considering its pharmacological properties, insulin therapy is the first-line drug therapy for the treatment of GDM [8]. It is considered as the gold standard therapy in patients who are unable to control their glycemia with lifestyle measures [10]. Requirements of insulin differ from woman to woman, with some requiring lower doses while others may need significantly higher doses [8]. However, use of insulin is associated with several limitations including need for injections, pain at the site of injection and cost of treatment.

With this background, clinicians are always in search of an alternative option. Although several oral agents are available in the market, their usage in pregnancy is controversial. Metformin crosses the placenta, but its usage is not associated with significant concerns in newborns [4,10]. Some data is available on the use of glyburide and acarbose in gestational diabetes mellitus [11]. According to a meta-analysis, glyburide is inferior than metformin and insulin in GDM [12].

Acarbose is an alpha glucosidase inhibitor which reduces absorption of carbohydrates from the small intestine by inhibiting breakdown of disaccharides and oligosaccharides. This helps in reducing the risk of postprandial hyperglycemia. Considering minimal absorption in the circulation, acarbose seems to be a promising option for the treatment of gestational diabetes mellitus. Currently, the ACARB-GDM-a phase III prospective, multi-center, non-inferiority, randomized trial is on-going to study acarbose and prandial insulin as a treatment of GDM [13]. There is lack of data from well-designed clinical trials in gestational diabetes mellitus among patients from India.

The objective of this study was to evaluate fetomaternal outcomes in women with GDM treated with insulin versus acarbose.

## Materials and methods

In this prospective, open label and controlled study, pregnant women attending the obstetrics outpatient department between January 2017-January 2018 who underwent 75 gm oral glucose tolerance test and those having fasting plasma glucose more than 92 mg/dl, one hour postprandial glucose level more than 180 mg/dl and two hour postprandial glucose level more than 153 mg/dl were included in the study. Patients with multifetal gestations, pre-gestational diabetes (type 1 or type 2 diabetes mellitus), diabetes diagnosed before 10 weeks of gestation or GDM patients with other chronic diseases and gestational age at delivery less than 20 weeks were excluded. 

Patients treated with acarbose and insulin were observed for outcomes until six weeks after delivery. Maternal outcomes i.e. incidence of recurrent genitourinary infections, pre-eclampsia, premature rupture of membranes, gestational age at the time of delivery and modes of delivery were compared between two groups. Post-operative/delivery random blood glucose, fasting blood glucose and postprandial glucose at day seven and after six weeks was checked in mothers treated with both the drugs. Glycosylated haemoglobin was examined post six weeks after delivery in both groups. In new born babies, mean (SD) birth weight was calculated. Mean (SD) random blood glucose levels were checked in babies in the both groups. Incidence of babies with birth weight of more than 3500 g, intrauterine growth retardation, birth trauma, birth asphyxia, neonatal jaundice, admission to the special new born care unit and congenital anomaly was calculated and compared between mothers with gestation diabetes treated with insulin versus acarbose.

Continuous data is presented as mean and standard deviation whereas categorical data is presented as number and percentages. Chi-square test and students unpaired ‘t’ test were used for comparison of categorical data and continuous data between two groups respectively. P value of less than 0.05 was considered significant.

## Results

A total of 100 patients (acarbose group n=50 and insulin n=50) were included in the study. The mean age of the patients treated with insulin and acarbose was 28.52 years and 26.26 years respectively (p=0.020). There was no significant difference in body mass index of patients between two groups (p=0.157; Table 1)

**Table 1:** Baseline characteristics of patients in both groups

	Insulin (n=50)	Acarbose (n=50)	P value
Mean (SD) age in years	28.52 (5.09)	26.26 (4.4)	0.020
Mean (SD) BMI (kg/m^2^)	26.50 (3.20)	25.56 (3.28)	0.157
Primigravida n (%)	14(28)	18(36)	0.39
Multigravida n (%)	36(72)	32(64)	
Primipara n (%)	20(40)	27(54)	0.16
Multipara n (%)	30(60)	23(46)	
Hypertension	5 (10%)	9 (18%)	0.249
Thyroid disease	6 (12%)	7 (14%)	0.766
Family history of diabetes n (%)	14 (28%)	12 (24%)	0.648
History of gestational diabetes mellitus n (%)	16 (32%)	13 (26%)	0.50
Mean (SD) gestational age at the time of diagnosis in weeks	16.86 (11.71)	16.08 (2.94)	0.245
Mean (SD) blood glucose after 2 hours of 75 gm glucose tolerance test (mg/dl)	166.68 (11.71)	163.72 (6.35)	0.119
Mean (SD) fasting blood glucose at the time of diagnosis (mg/dl)	110.44 (7.80)	111.82 (7.56)	0.372
Mean (SD) postprandial blood glucose (mg/dl) at the time of diagnosis	164.14 (16.31)	162.66 (16.31)	0.560

There was no significant difference between the two groups in terms of past history of hypertension (p=0.249), thyroid disease (p=0.766), family history of diabetes (p=0.648) or history of gestational diabetes mellitus (p=0.50). A total of 14 (28%) and 18 (36%) of patients in the insulin and acarbose groups respectively were primigravida (p= 0.39), whereas 30 (60%) and 23 (46%) patients in the insulin and acarbose group were multiparous respectively (p=0.16). There was no significant difference in the mean gestational age at diagnosis between patients in the insulin group versus those in acarbose group (16.86 vs 16.08 weeks; p=0.245). Similarly, there was no significant difference in the mean blood glucose after two hours of 75 g of glucose tolerance test (p=0.119), mean fasting blood glucose at diagnosis (p=0.372) and mean postprandial blood glucose (p=0.560) at the time of diagnosis.

**Figure 1:**
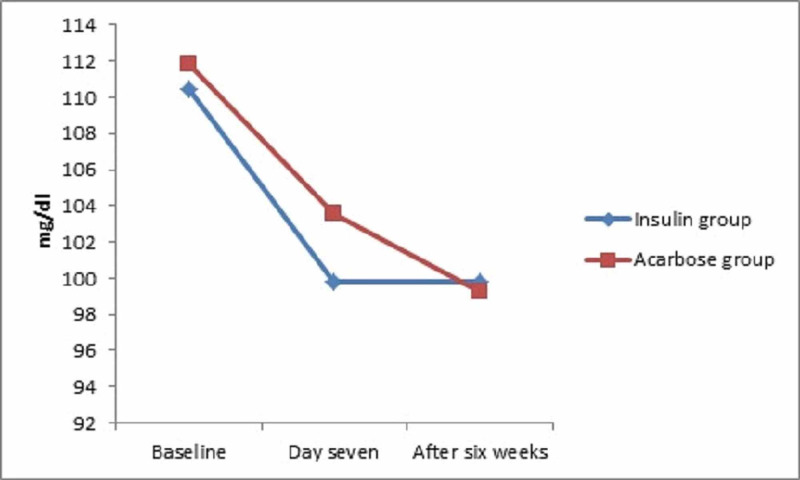
Reduction in mean fasting blood glucose in patients with gestational diabetes treated with insulin and acarbose

There was no significant difference in the reduction of mean (SD) fasting blood glucose level at day seven [99.80 (15) vs 103.58 (10.78); p=0.15) and after six weeks [99.84 (14.78) vs 99.30 (11.56); p=0.83] between the insulin and acarbose groups respectively (Figure 1).

**Figure 2:**
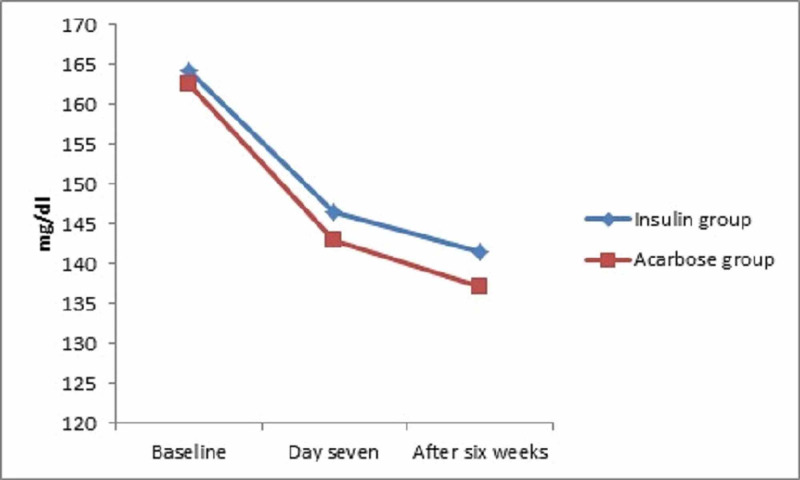
Reduction in mean post-prandial blood glucose in patients with gestational diabetes treated with insulin and acarbose

There was no significant difference in reduction in the mean (SD) postprandial blood glucose level at day seven [146.52 (32.12) vs 142.92 (16.38); p=0.48) and after six weeks [141.44 (21.37) vs 137.08 (14.54); p=0.23] between the insulin and acarbose groups respectively (Figure 2).

Similarly, mean (SD) HbA1c levels in the insulin group [6.50 (0.41)] and acarbose group [6.42 (0.30)] were similar (p=0.82). During the antenatal period, 10 (20%) of the patients treated with insulin and 9 (18%) of the patients treated with acarbose developed recurrent infections (p=0.64). A total of 14 (28%) of the patients treated with insulin and 12 (24%) of the patients treated with acarbose developed preeclampsia (p=0.64). Preterm-premature rupture of membranes (PPROM)/premature rupture of membranes (PROM) was seen in 16 (32%) of the cases treated with insulin versus 20 (40%) of the cases treated with acarbose (p=0.40) (Table 2).

**Table 2:** Maternal outcomes in two study groups

	Insulin (n=50)	Acarbose (n=50)	P value
Recurrent genitourinary infections	10 (20%)	9 (18%)	0.79
Preeclampsia	14 (28%)	12 (24%)	0.64
Preterm-Premature rupture of membranes (PROM)/Premature rupture of membranes (PROM)	16 (32%)	20 (40%)	0.40
Delivery at mean (SD) gestational weeks	36.93 (2.71)	38.36 (1.68)	0.002
Lower (uterine) segment caesarean section	28 (56%)	33 (66%)	0.55
Vaginal delivery	19 (38%)	14 (28%)	0.55
Instrumental delivery	3 (6%)	3 (6%)	0.55

The mean (SD) duration of gestational week at the time of delivery in the insulin treated group and acarbose treated groups was 36.93 (2.71) and 38.36 (1.68) weeks respectively (p=0.002). There was no significant difference in the modes of delivery between two groups for lower (uterine) segment caesarean section (p=0.55), vaginal delivery (p=0.55) and instrumental delivery (p=0.55). There was no difference in the mean (SD) post-operative random blood glucose in insulin treated group versus acarbose treated group [113.38 (25.85) vs 113.66 (29.48) mg/dl; p=0.96].

There was no significant difference in the mean (SD) birth weight of babies born to mothers treated with insulin versus acarbose [2580 (720.02) vs 2744 (577.17) grams; p=0.21]. Similarly, there was no significant difference in the mean (SD) random blood glucose levels in babies born to mothers after insulin treatment versus those born to mothers treated with acarbose [58.92 (12.08) vs 57 (14.06) mg/dl); p=0.46]. A total of 6 (12%) babies in both groups had random blood glucose less than 45 mg/dl after delivery.

A total of 2(4%) and 4 (8%) babies in the insulin group and acarbose group had birth weight of more than 3500 g respectively. Intrauterine growth retardation was observed in 9 (18%) and 7 (14%) babies in the insulin and acarbose group respectively.

**Figure 3:**
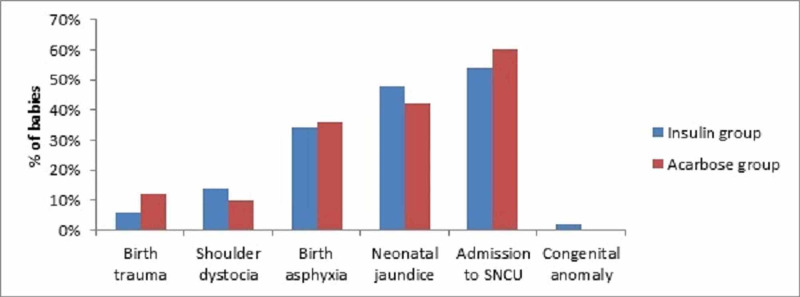
Neonatal complications in patients with gestational diabetes mellitus treated in insulin and acarbose SNCU: Special new born care unit

There was no difference in the incidence of birth trauma [3 (6%) vs 6 (12%); p=0.29], shoulder dystocia [ 7 (14%) vs 5 (10%); p=0.53], birth asphyxia [17 (34%) vs 18 (36%); p=0.83], neonatal jaundice [22 (44%) vs 21 (42%); p=0.84], admission to special new born care unit [27 (54%) vs 30 (60%), p=0.54] and congenital anomaly [1 (2%) vs 0%; p=0.31] between patients treated with insulin versus acarbose respectively (Figure 3).

## Discussion

It is important to recognize GDM and treat it effectively to avoid complications in mothers as well as newborns. Currently, diet and insulin are important and the commonly used options in the treatment of GDM [4]. Insulin therapy is considered as the gold standard and treatment of choice in GDM [14,15]. It is advised to women not controlled on diet [16]. While insulin and lifestyle modifications are insufficient to provide effective relief, certain oral antihyperglycemic drugs can be considered as alternative treatment options [17]. In this study, we compared outcomes of GDM and newborns in mothers treated with insulin and acarbose.

In our study, all baseline characteristics including body mass index, comorbidities, family history of diabetes, history of GDM among patients, mean fasting glucose and postprandial blood glucose at the time of diagnosis of GDM in acarbose and insulin group were similar except mean age. Mean age in patients in insulin group was more than those in the acarbose group.

A small case series of six patients reported normalization of fasting and postprandial glucose levels in all cases. All pregnancies were uneventful and the newborns were normal [18]. Berini et al. compared neonatal results in 70 mothers with GDM treated with insulin, glyburide and acarbose [11]. There was no difference in the maternal characteristics between groups and glucose control was not achieved in higher percentage of patients with acarbose than glyburide. There was no difference in fasting and post-prandial glucose levels between the three groups [11]. In our study, there was no significant difference in the reduction of fasting and postprandial blood glucose level at day seven and after six weeks between insulin and acarbose groups. Similarly, mean HbA1c levels in insulin group and acarbose group at six weeks were similar.

In our study, there was no significant difference in the insulin and acarbose groups for the rates of recurrent infections, or preeclampsia. Similarly, the rates of premature rupture of membranes (PROM) were similar between two groups. A meta-analysis of oral antidiabetic drugs and insulin in GDM reported highest incidence of preeclampsia and hyperbilirubinemia with glyburide [19]. A Cochrane review reported increased risk of hypertensive disorders of pregnancy with insulin as compared to oral anti-diabetic drugs [20]. Metformin (plus insulin when required) is associated with low incidence of pregnancy induced hypertension [19]. A Cochrane analysis reported no difference between insulin and oral anti-diabetic drugs for the risk of preeclampsia [20]. According to the results of another meta-analysis, insulin had significantly higher risk of preeclampsia than metformin [21].

In our study, there was no significant difference in the modes of delivery between two groups. The mean duration of gestational week at the time of delivery in the insulin treated group and acarbose treated groups was similar. A Cochrane review also reported no difference between insulin and oral anti-diabetic drugs for the risk of birth by caesarean section [20]. In our study, there was no difference in the incidence of complications during delivery including birth trauma, shoulder dystocia, birth asphyxia or neonatal jaundice between patients treated with insulin versus acarbose respectively. However, birth trauma was mostly seen in instrumental vaginal delivery and some of the lower segment caesarean section cases. Injuries were mostly due to blades of forceps. In one case, there was a fractured humerus and the baby was delivered by assisted breech. Birth asphyxia was due to meconium aspiration and macrosomic babies leading to a prolonged second stage of labour.

We did not observe significant difference in the mean birth weight of babies born to mothers treated with insulin versus acarbose. A total of 4% and 8% babies in the insulin group and acarbose group had birth weight of more than 3500 g respectively. In another study, there was no difference in newborn weight in mothers with GDM treated with insulin, glyburide and acarbose [11]. Significant increase in birth weight and gestational age at delivery has been reported with insulin as compared to metformin [21]. In a meta-analysis of oral antidiabetic drugs and insulin, shortest gestational age at delivery and lowest mean birth weight has been reported with glyburide [19]. Metformin (plus insulin when required) is associated with low incidence of low birth weight and low gestational age at delivery [19].

Rate of macrosomia has been reported to be higher with glyburide than acarbose [11]. A meta-analysis of oral antidiabetic drugs and insulin in GDM reported glyburide to have highest incidence of macrosomia [19]. Metformin is associated with low incidence of macrosomia [19]. According to the results of a meta-analysis, insulin had significantly higher risk macrosomia than metformin [21].

In our study, there was no significant difference in the mean random blood glucose levels in babies born to mothers after insulin treatment versus those born to mothers treated with acarbose. Similarly, the rate of new-born babies with random blood glucose less than 45 mg/dl after delivery was similar in both groups. A meta-analysis of oral antidiabetic drugs and insulin in GDM reported highest incidence of neonatal hypoglycemia with glyburide [19]. In a systematic review, insulin was reported to have higher rates of neonatal hypoglycemia than metformin [22]. In another study, neonatal hypoglycemia was observed in more cases with glyburide than insulin and acarbose [11]. A meta-analysis of oral antidiabetic agents like insulin reported lowest risk of neonatal hypoglycemia with acarbose [19]. Neonatal hypoglycemia is one of the important concerns with insulin in gestational diabetes. A study reported 33% and 35% incidence of mild and severe hypoglycemia with insulin respectively [23]. However, in a Cochrane review, there was no evidence of a clear difference between insulin and oral antidiabetic therapy [20]. In another meta-analysis, glyburide had a higher increase of neonatal hypoglycemia compared to insulin [21]. According to the results of a meta-analysis, insulin had significantly higher risk of neonatal hypoglycemia than metformin [21].

In a systematic review, rate of congenital malformations was similar with insulin and oral drugs [22]. In our study, congenital anomaly was seen only in one case of the insulin treated group in form of congenital talipes equinovarus. No cases of congenital anomaly were seen in acarbose treated group. 

In a study amongst metformin, glyburide and insulin, insulin had the highest incidence of neonatal intensive care unit admission [19]. Metformin has been reported to be associated with low incidence of respiratory distress syndrome [19]. According to the results of a meta-analysis, insulin had significantly higher risk of neonatal intensive care unit admission than metformin [21]. In our study, there was no difference in admission to special new born care unit between patients treated with insulin versus acarbose. The causes of admission to special new born care unit were mostly due to neonatal jaundice, birth asphyxia, hypoglycaemia, preterm delivery and intrauterine growth retardation. All cases of neonatal jaundice had unconjugated hyperbilirubinemia which included both physiological and pathological jaundice.

Acarbose is associated with intestinal discomfort [18]. In our study, acarbose was well tolerated by the patients without any major side effects. Overall, our study suggests promising role of acarbose in patients with GDM. The results of an on-going multicentre study ACARB-GDM will provide more insights on the outcomes in patients with GDM [13].

Our study has some limitations. It was conducted at a single center and the relatively small sample size are the other limitations. Larger, multicentre studies are required to confirm our observations.

## Conclusions

In our study, there were no significantly adverse neonatal or maternal outcomes or congenital anomalies in the acarbose treated group of patients with GDM. The glycemic control in patients with GDM was comparable between groups treated with acarbose and insulin. Our preliminary results suggest that acarbose is an attractive option for treatment of GDM. More studies involving large number of patients are required for the recommendation of use of acarbose in GDM.
